# Mental Health and Resilience in Eritrean and Somali Refugees in Switzerland: A Cross-Sectional Study

**DOI:** 10.3389/ijph.2026.1608308

**Published:** 2026-03-11

**Authors:** Jennifer Giovanoli Evack, Charles Abongomera, James Okuma, Johanna Kurscheid, Yeabio Melake, Afona Chernet, Tesfalem Ghebreghiorghis, Anna Verjans, Fiona Vanobberghen, George Abongomera, Jan Fehr, Naser Morina, Daniel H. Paris

**Affiliations:** 1 Swiss Tropical and Public Health Institute, Allschwil, Switzerland; 2 University of Basel, Basel, Switzerland; 3 Department of Public and Global Health, Epidemiology, Biostatistics and Prevention Institute, University of Zurich, Zurich, Switzerland; 4 Department of Consultation-Liaison Psychiatry and Psychosomatic Medicine, University Hospital of Zurich, University of Zurich, Zurich, Switzerland

**Keywords:** anxiety, somatic disorder, depression, displaced people, post-traumatic stress disorder

## Abstract

**Objectives:**

In this study, we determined the frequency of clinically relevant mental health conditions among Eritrean and Somali refugees in Basel and Zurich, Switzerland and assessed their levels of resilience.

**Methods:**

A cross-sectional study among refugees aged ≥16 years involved validated questionnaires, screening for symptoms of post-traumatic stress disorder (PTSD), somatic disorders, anxiety, depression, and assessments of alcohol use and resilience.

**Results:**

The 102 participants were young (median age 34 years), Eritrean (N = 88; 86%), males (N = 62), with long periods of staying in Switzerland (median 8.6 years). Almost three-quarters (N = 69/99; 70%) had high resilience scores (median 86). We found low frequencies of moderate or severe symptoms of PTSD (7%), anxiety (0%) and depression (1%). However, symptoms for somatic disorders (18%) and harmful levels of alcohol use (12%) were more common.

**Conclusion:**

While the overall levels of resilience are impressively high, a large proportion of participants exhibited symptoms of somatic disorders and harmful levels of alcohol use - well beyond the early adaptation phase. This highlights the need for long-term mental healthcare beyond the time of arrival to ensure their wellbeing.

## Introduction

Recent geopolitical events have led to a surge in human migration. The United Nations High Commission for Refugees (UNHCR) reports that in 2023, 117.3 million people were forcibly displaced worldwide, an increase of 8%, (8.8 million people) since 2022 – these numbers have been increasing yearly over the past 12 years [1]. This has revealed significant issues concerning the health of migrants, especially mental health and lack of access to adequate healthcare during the migration journey, and during integration in host countries [2].

Migration itself is an established social determinant of health, affecting the physical, mental and social wellbeing and overall health of refugees and migrants [3]. The migration process is dynamic and complex with challenges to health and wellbeing, as well as traumatic events that may occur across all phases - pre-migration, transit, and arrival and integration in the host country. Circumstances in the country of origin, such as political instability, conflict, and environmental factors influence migrants’ baseline health [3]. During the migration journey, lack of access to healthcare and exposure to traumatic events such as conflict, physical and sexual violence, detention, torture, discrimination, exploitation and loss or separation from family can further negatively affect mental health [4, 5]. In the host country, circumstances such as the political environment, discrimination, complexities associated with acculturation, fear of deportation and long waits for asylum application decisions can lead to a persistent state of unpredictability and uncontrollability of one’s life [3, 6, 7]. Financial hardship and economic stressors can augment these difficulties, as the worry of meeting basic daily needs contributes to a sense of uncertainty about the future [7].

Individuals who flee their country seeking legal refugee status are considered asylum seekers. After their individual situation has been assessed by the host country’s government, and it has been determined that there is a threat to safety due to persecution, armed conflict, or violence, they are given legal refugee status and considered refugees, as per the 1951 refugee convention [8]. Immigration from Eritrea and Somalia into Switzerland has been ongoing for the last 2 decades due to political and economic instability and climatic issues such as drought and floods [9–11]. In 2022, Eritreans and Somalis were the 3^rd^ and 6^th^ largest groups to submit applications for asylum in Switzerland [12] and over 40% of all refugees in Switzerland originated from Eritrea or Somalia, with Eritreans representing the largest group [13]. Migrants from the Horn of Africa to Europe typically travel along the northern migration route through Sudan, Libya and the Mediterranean. This route is associated with extreme risks, including arbitrary detention, torture, extortion, human trafficking, murder, sexual and other severe violence and death. It also involves the Mediterranean sea crossing, the deadliest migration route, where many drownings occur [10, 14, 15]. Psychological trauma experienced during migration, post migration stressors and triggers of prior traumatic experience can have significant and prolonged impact on mental health [4, 6].

The World Health Organization’s (WHO) review of global health data, along with several meta-analyses, show a high burden of mental health conditions among refugees and migrants, with reported prevalences of 32% for depression, 11% for anxiety and 32% for post-traumatic stress disorder (PTSD), although estimates vary across studies [6, 16, 17]. Evidence indicates that migrants -particularly asylum seekers and refugees- experience higher rates of depression, anxiety, PTSD, postpartum depression and psychosis than the general population [3, 6].

In Switzerland, studies similarly report substantial mental health needs. One investigation among refugees found rates of depression, anxiety and PTSD of 42%, 39% and 41%, respectively [18]. Another study focusing on recently arrived Eritrean migrants in Switzerland, reported prevalences of symptoms of depression, anxiety and somatic disorders of 15%, 10% and 10%, respectively, and nearly half of respondents screened positive for symptoms of PTSD (49%) [19]. Among migrants -including undocumented individuals- who had been living in Geneva for 5 years or more, prevalences of symptoms of anxiety (36%), depression (45%) and sleep disturbance (23%) were documented [20].

Harmful levels of alcohol and substance use have been observed among forced migrants [21]. Mental ill-health and harmful alcohol or substance use are closely intertwined [22]; alcohol may be used as a coping mechanism for trauma experienced before, during and after migration, but can also predispose individuals to further health risks, including liver cirrhosis and cancer [22]. Conversely, resilience has been shown to mitigate the effects of trauma and mental health conditions [23]. Only a few studies have investigated levels of resilience in migrants, including a study of Eritrean refugees in Switzerland, which found high levels of resilience in this population (60%) [19].

Despite the importance of mental health in refugees, there are few studies in Switzerland evaluating the prevalence of mental health conditions and/or their symptoms as well as substance use in refugees, especially in Eritrean and Somali populations [17–20, 24]. The reported findings vary by the type of questionnaires and the cut-offs used for assessments. Factors like mental health diagnostic criteria, migrant group (country of origin, type of migrant–asylum seeker, refugee, illegal migrant, labor migrant), host country and duration of residence there, socioeconomic status and integration policies play important roles [4, 6, 18, 25]. Consequently, the current landscape of epidemiological data concerning the mental health of refugees in Switzerland is limited and there is a clear need for robust evidence to inform migration health policies.

The study aimed to systematically assess the frequency of clinically relevant mental health conditions in Eritrean and Somali refugees beyond the early adaptation phase to inform migration health policies in Switzerland. Specifically, we determined the proportion of symptoms of PTSD, somatic disorder, anxiety, depression and unhealthy alcohol use as well as level of resilience in the Eritrean and Somali communities in Basel and Zurich, Switzerland. We hypothesized that mental health conditions, harmful or hazardous drinking and levels of resilience were substantially underappreciated in this population.

## Methods

### Study Design and Setting

This cross-sectional study was conducted by the Swiss Tropical and Public Health Institute (Swiss TPH) in Basel, Switzerland in collaboration with the Department of Public and Global Health at the Institute of Epidemiology, Biostatistics and Prevention (EBPI) at the University of Zurich, Zurich, Switzerland.

### Participants and Recruitment

From January 2022 to March 2023, we recruited Eritrean and Somali refugees in Basel and Zurich, Switzerland who were aged 16 years or older and provided written informed consent to participate in the study. Individuals who were critically ill (requiring emergency treatment) were excluded. Participation was voluntary, travel expenses were reimbursed and participants were free to withdraw at any time.

Recruitment of participants was achieved with the assistance of organizations and key community leaders working with migrants. Potential participants were asked to participate in a study about their health and wellbeing, with the benefit that they would receive a health check, free of charge (including screening for some micronutrient and non-communicable diseases). In addition, research assistants canvassed locations known to be frequented by refugees/asylum seekers, such as restaurants, markets, and on the street, approaching potential participants directly and inviting them to take part in the study. These four trained research assistants from the Eritrean and Somali diasporas, who spoke the local language (Tigrinya for Eritreans and Somali for Somalis), recruited the study participants and conducted the interviews. The research assistants were trained on study protocols, questionnaires (in Tigrinya, Somali and English) and psychological first aid. The research assistants explained the study to the potential participants and scheduled interviews.

### Data Sources and Measurement of Variables

The interviews were conducted during the work week in a quiet, private, dedicated room. Data were collected on digital tablets via electronic case report forms (eCRFs) developed on the Research Electronic Data Capture (REDCap) platform [26, 27]. The following demographic data were collected: age, nationality, sex, marital status, education level, former and current employment, ethnicity, religion, length of time in Switzerland and residency.

The primary outcomes were frequencies of symptoms of mental health conditions (PTSD, somatic disorders, anxiety and depression), harmful alcohol-use and levels of resilience. Validated questionnaires were used and had been translated from English into Tigrinya and Somali and subsequently back translated for quality control. The questionnaires were administered in the preferred language of the participant. Three additional questions were included asking whether participants had experienced traumatic events, imprisonment or difficulties during migration. If participants perceived their imprisonment as traumatic, this may have been captured in the traumatic event question, but we did not ask participants whether their imprisonment was traumatic. Participants who did not view their imprisonment as traumatic would not necessarily have answered “yes.” Participants with moderate and severe symptom scores were advised to go to a physician for further evaluation.

#### Symptoms of PTSD

The PTSD checklist (PTSD-CL-S) [28] has 17 questions to measure the 17 symptoms of PTSD in the Diagnostic and Statistical Manual of Mental Disorder IV [DSM- IV], focusing on symptoms occurring in the last month related to stressful life experiences. Each item is scored from 1 to 5 (1 = not at all to 5 = extremely), resulting in an overall score between 17 and 85. Severity cut-off scores are 17–29 (little to no severity) and ≥30 (moderate to high severity) [28, 29]. Scores of ≥30 were considered indicative of potentially clinically relevant symptoms. This version of the PTSD-CL-S has been used in this population before [19] and has good internal consistency, with a Cronbach’s alpha above 0.75 [30].

#### Symptoms of Somatic Disorders

The Patient Health Questionnaire (PHQ) 15 (PHQ-15) [31, 32] estimates severity of somatic symptoms through 15 items which ask patients to rate how much they have been bothered by the 15 main symptoms of somatic disorders over the previous 4 weeks. Each question is scored from 0 (not bothered) to 2 (bothered a lot) with a total score ranging from 0 to 30 for women and 0–28 for men (omitting the menstrual pain question). Cut-off scores of 5, 10 and 15 are used to indicate mild, moderate and severe symptom severity, however, scores of ≥10 flag those with a clinically significant condition and ≥15 flag those for whom treatment is warranted [33]. In this investigation, scores of 10 or above were considered indicative of potentially clinically relevant symptoms. The PHQ-15 has been used in large cohort studies, has good internal consistency (Cronbach’s alpha of 0.80) [34] and has been used in this population of migrants [19, 34, 35].

#### Symptoms of Anxiety

The 7-item General Anxiety Disorder (GAD-7) [31, 32], explores common symptoms of anxiety. It is scored from 0 to 3 (0 = not at all to 3 = nearly every day), with overall scores ranging from 0 to 21. As with the PHQ-15, cut-off scores representing mild [5], moderate [10] and severe [15] severity of symptoms are used [33] with scores ≥10 and ≥15 as flags and scores of ≥10 were indicative of potentially clinically relevant symptoms. GAD-7 is internally consistent with Cronbach’s alpha of 0.80 [34]. It is a commonly used tool in research settings and has previously been used in Eritrean migrants [19, 34, 36, 37].

#### Symptoms of Depression

The PHQ-9 [31, 32] is a tool for assessing common depressive symptoms. It consists of nine questions scored from 0 to 3, as per the GAD-7, with overall scores ranging from 0 to 27. Severity of symptoms are assessed by cut-off scores of 5, 10, 15 and 20 representing mild, moderate, moderately severe and severe. Scores 10 or above were considered indicative of potentially clinically relevant symptoms [33]. This tool has good internal consistency (Cronbach’s alpha of 0.86–0.89) [34] and has been used extensively in both clinical settings and research, specifically having been used in this population in Switzerland before [19, 34].

#### Alcohol Consumption

The Alcohol Use Disorders Identification Test (AUDIT) [38] is used to identify harmful or hazardous patterns of alcohol consumption. There are 10 questions scored from 0, never consume, to 4 indicating high consumption, with a total score ranging from 0 to 40. A score of 0 indicates no problems with alcohol (including those who do not drink), 1-7 indicates low risk alcohol consumption, a score between 8 and 15 suggests hazardous drinking, while a score of 16–19 alerts to high risk or harmful drinking and 20 or above flags possible alcohol dependence [38]. Scores 8 or above were considered indicative of potentially clinically relevant harmful alcohol use. The AUDIT questionnaire has been used extensively in clinical and research settings, across cultures and in this study population in Switzerland [19, 39–41]. The questionnaire has high internal consistency with Cronbach’s alpha of approximately 0.80 being consistently reported [39, 42].

#### Resilience

The Resilience Scale 14 (RS14) measures resilience through a 14-item questionnaire using a scale of 1–7 (1 = not at all, to 7 = nearly every day) with scores ranging from 14 to 98. A score of <65 indicates low resilience, 65–81 moderate resilience and ≥82 high resilience [43, 44]. This questionnaire has been used in a variety of populations, including migrants and has good internal consistency (Cronbach’s alpha coefficient ranging between 0.76 and 0.96) [19, 25].

### Sample Size and Statistical Analysis

The sample size of 200 participants was determined by the feasibility to recruit migrants and the study budget. Due to the COVID-19 pandemic related travel restrictions, the number of new refugees arriving in Switzerland were remarkably reduced and recruitment was stopped after 102 participants. Median and interquartile ranges (IQR) of continuous variables, and numbers and percentages of categorical variables were used to describe sociodemographic characteristics and frequency of mental health symptoms, alcohol use and resilience. Cronbach’s alpha (α) for internal consistency was calculated for all mental health questionnaire tools, with α ≥0.65 indicating acceptable internal consistency [45]. We conducted descriptive sub-group analyses to investigate mental health symptoms, alcohol use and resilience by age group (under 35 years or 35 years and older), sex and those with history of imprisonment or other difficulties during migration. Analyses were performed using Stata version 18 (StataCorp LP, College Station, Texas, USA).

### Ethics

Ethical approval for the study was obtained from EKNZ, Basel, Switzerland (2020-02154). All procedures were in accordance with the ethical standards of the Swiss TPH, EBPI, the Ethikkommission Nordwest- und Zentralschweiz (EKNZ) and with the 1964 Helsinki declaration and its later amendments or comparable ethical standards.

## Results

### Study Population

**FIGURE 1:**
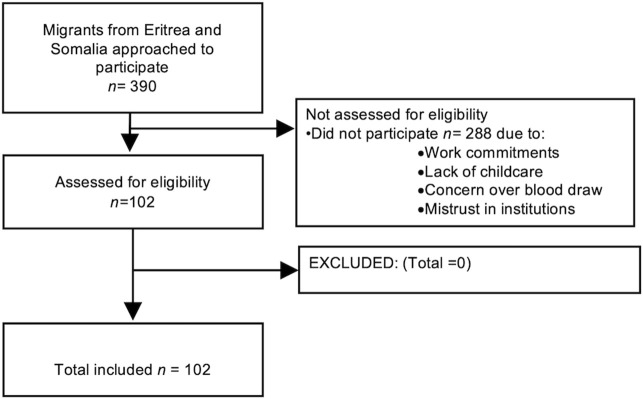
Flow diagram of recruitment of Eritrean and Somali refugees. Mental health symptoms in Eritrean and Somali refugees, Switzerland, 2022–2023.

A total of 390 potential participants were contacted to participate in the study (Figure 1).

Of the 390 potential participants, 288 participants did not come for assessment of eligibility to participate in the study and 102 participants were included in the study. There is no disaggregated data for reasons for non-participation, but not having time to participate due to work commitments or childcare, disinterest and mistrust in the research project were mentioned.

### Socio-Demographic Health Characteristics

Participants were relatively young with a median age of 34 years (IQR: 26–47), most were Eritrean (N = 88/102; 86%), male (N = 62/102; 61%), married (N = 52/102; 51%), living in Switzerland for a median of 8.6 years (IQR: 7.0–11.4) with resident status (N = 99/102, 97%) (Table 1).

**TABLE 1:** Socio-demographic characteristics of Eritrean and Somali refugees in Basel and Zurich, Switzerland (2022–2023).

Characteristics	N = 102
Age (years) [median (IQR)] n = 102 Age categories (years) [n (%)] n = 102 17–24 25–34 35–44 ≥45	34 (26–47) 13 (13%) 41 (40%) 17 (17%) 31 (30%)
Nationality, [n (%)] n = 102 Eritrean Somali	88 (86%) 14 (14%)
Sex, [n (%)] n = 102 Male Female	62 (61%) 40 (39%)
Marital status, married [n (%)] n = 102 Married Never married Separated/divorced/Widowed	52 (51%) 34 (33%) 16 (16%)
Highest education level, [n (%)] n = 101 None Primary Secondary and above	7 (7%) 46 (46%) 48 (48%)
Previous occupation, [n (%)] n = 100 Unemployed Student Farmer Employed Homemaker	5 (5%) 41 (41%) 10 (10%) 38 (38%) 6 (6%)
Current occupation, [n (%)] n = 100 Unemployed Student Employed Housewife	20 (20%) 8 (8%) 52 (52%) 20 (20%)
Ethnicity, [n (%)] n = 101 Tigrigna Somali Others	84 (83%) 14 (14%) 3 (3%)
Religion, [n (%)] n = 102 Orthodox Muslim Others	73 (71%) 19 (19%) 10 (10%)
Years living in Switzerland (years) [median (IQR)] Years living in Switzerland (years) [n (%)] n = 96 0 – <5 5–9 ≥10	8.6 (7.0–11.4) 11 (11%) 48 (50%) 37 (39%)
Refugee status [n (%)] n = 102 In asylum center Resident status	3 (3%) 99 (97%)

Results are number, column %, median and interquartile range of those with non‐missing data, missing data indicated in N for each variable. IQR: interquartile ranges. Mental health symptoms in Eritrean and Somali refugees, Switzerland, 2022–2023.

### Proportion of Symptoms of Mental Health Conditions and Associated Factors

All questionnaires demonstrated acceptable internal consistency (α ≥ 0.65).

#### History of Trauma, Imprisonment and Other Difficulties During Migration

Nearly one-third of participants (N = 32/100) reported ever experiencing a traumatic event. A history of imprisonment was reported among 40% (N = 40/99) of participants, while difficulties during migration were reported by 10% (N = 10/99) (Table 2).

**TABLE 2:** Mental health symptom scores among Eritrean and Somali refugees in Basel and Zurich in Switzerland (2022–2023).

Mental health questionnaire	N = 102
Experience any traumatic event, yes [n (%)] n = 100 History of imprisonment during migration, [n (%)] n = 99 Other difficulties during migration, [n (%)] n = 99	32 (32%) 40 (40%) 10 (10%)
PTSD PTSD-CL-S score [median (IQR)] PTSD-CL-S categories [n (%)] n = 99 0 no traumatic events 17–29 little to no severity ≥30 moderate to high severity Internal consistency (α)	0 (0–17) 68 (69%) 24 (24%) 7 (7%) 0.91
Somatic disorder PHQ-15 score [median (IQR)] PHQ-15 categories [n (%)] n = 97 0–4 none to minimal 5–9 mild 10–14 moderate ≥15 severe Internal consistency (α)	3 (1–8) 65 (67%) 14 (14%) 14 (14%) 4 (4%) 0.84
Anxiety GAD 7 score [median (IQR)] GAD 7 categories [n (%)] n = 98 0–4 none to minimal 5–9 mild 10–14 moderate ≥15 severe Internal consistency (α)	1 (0–3) 89 (91%) 9 (9%) 0 (0%) 0 (0%) 0.65
Depression PHQ-9 score [median (IQR)] PHQ-9 categories [n (%)] n = 99 0–4 none to minimal 5–9 mild 10–14 moderate 15–19 moderately severe ≥20 severe Internal consistency (α)	1 (0–3) 89 (90%) 9 (9%) 1 (1%) 0 (0%) 0 (0%) 0.65
Resilience RS14 score [median (IQR)] RS14 categories [n (%)] n = 99 45–64 low resilience 65–81 moderate resilience ≥82 high resilience Internal consistency (α)	86 (80–91) 3 (3%) 27 (27%) 69 (70%) 0.80
Alcohol consumption Alcohol consumption, yes [n (%)] n = 100 AUDIT score [median (IQR)] AUDIT categories [n (%)] n = 100 0 no risk 1–7 low risk 8–15 risk/hazardous 16–19 high risk/harmful ≥20 possible alcohol dependence Internal consistency (α)	50 (50%) 0 (0–3) 50 (50%) 38 (38%) 11 (11%) 1 (1%) 0 (0%) 0.75

PTSD: post-traumatic stress disorder, PTSD-CL-C: post-traumatic stress disorder checklist (civilian), IQR: interquartile ranges PHQ: patient health questionnaire, GAD7: general anxiety disorder 7 questionnaire, RS: resilience scale 14 questionnaire, AUDIT: alcohol use disorder identification test. Results are number, column %, median and IQR, of those with non‐missing data, missing data rows are number and column % except where otherwise indicated. Mental health symptoms in Eritrean and Somali refugees, Switzerland, 2022–2023.

More men and those 35 years and older were imprisoned during migration than women and those under 35 years (49% of men, N = 30/61 versus 26% of women, N = 10/38; 49% 35 years and above, N = 23/47 versus 33% under 35 years, N = 17/52) (Supplementary Tables S1, S2).

#### Symptoms of PTSD, Somatic Disorder, Anxiety and Depression

The frequency of symptoms of PTSD were as follows: “no to little” severity of symptoms – 93% (N = 92/99); and moderate to high severity of symptoms – 7% (N = 7/99).

The frequency of moderate or severe symptoms of somatic disorders (PHQ-15) were elevated (18%; N = 18/97), as opposed to anxiety (GAD-7) and depression (PHQ-9), which were low in this population at 0% (N = 0/98) and 1% (N = 1/98), respectively (Table 2). More women than men had symptoms of somatic disorders (29%, N = 11/37; 11%, 7/60) (Supplementary Table S1). There were no differences found when the data was stratified by age or those with a history of imprisonment or other difficulties during migration (Supplementary Tables S2, S3). PTSD, anxiety and depression were not included in the sub-analysis as the proportions of those with symptoms were too low.

#### Alcohol Use

More than half of the participants had ever consumed alcohol (50%, N = 50/100) and 12% (N = 12/100) had AUDIT scores suggestive of hazardous/harmful consumption. More men had ever consumed alcohol (61%, N = 37/61% vs. 33%, N = 13/39) and were more commonly using alcohol in a harmful/hazardous way (20%, N = 12/61% vs. 0%, N = 0/39) than women (Supplementary Table S1). There were no other differences between subgroups (Supplementary Tables S2, S3).

#### Resilience

Nearly three quarters of the participants (N = 69/99; 70%) had high resilience scores (RS14) and those who had been imprisoned or had other difficulties during migration had greater frequency of high resilience than those who had not (80%, N = 36/45; 61% 33/54). There were no differences found between sex or age (Supplementary Tables S1–S3).

## Discussion

In this study, we determined the frequency of clinically relevant mental health symptoms and resilience in Eritrean and Somali refugees living in Basel and Zurich, Switzerland. In this study population with a longer integration period (median duration of stay after arrival was 8.4 years), we found low frequencies of moderate or severe symptoms of PTSD (7%), anxiety (0%) and depression (1%). However, there were elevated frequencies of symptoms of somatic disorders (18%) and harmful alcohol use (12%). The majority of refugees had high resilience scores (70%).

A comparison of our results to those of another investigation of Eritrean refugees in Basel who had recently arrived [19] reveal that the frequency of symptoms of mental health conditions in our sample were generally lower: PTSD (7% vs. 49%), anxiety (0% vs. 10.3%), depression (1% vs. 15%) and harmful alcohol use (12% vs. 18%). Although, a higher proportion of our study participants had high resilience scores (70% vs. 60%), the frequency of somatic disorders (18% vs. 10%) was higher in our study compared to the previous study [19].

There are various explanations for the differences in frequency of symptoms of mental health conditions in our study as compared to the study conducted by Chernet et al. [19]. First, the refugee’s duration of stay in Switzerland since arrival were remarkably different; in our study, the refugees had been living in Switzerland for about 9 years, whereas in the previous study, the refugees had only recently arrived (1–2 years). The duration of stay of our sample was relatively long because our study was conducted during the COVID-19 pandemic, when international border crossings were severely restricted. Subsequently, the number of new refugees arriving in Switzerland who could be recruited was severely limited. The refugee’s time since arrival can have a positive influence on mental health symptoms, as time allows healing from traumatic events [46]. We attempted an analysis of mental health symptoms by length of stay, but as only 11 individuals (11%) had been in Switzerland for less than 5 years, the analysis was not informative. In our study, we found that a large proportion of refugees had experienced traumatic events (32%), imprisonment (40%) and other difficulties (10%) during their journey. The study conducted by Chernet et al. [19], reported that 38% had been imprisoned and 58% had witnessed the death of a close person. Psychological trauma is one of the main causes of mental health conditions in refugees [47]. It is known that many refugees manifest severe mental health symptoms within the first years of arrival in the host country [48, 49]. Furthermore, in a subset of participants with shorter integration phases, who returned for follow-up 1 year later, most of the symptoms had decreased: anxiety (8% vs. 4%), depression (15% vs. 6%), PTSD (50% vs. 25%), except harmful alcohol use, which remained high (13%) [19].

During the first years of adaptation in Switzerland, refugees face post-migration stressors related to unemployment, integration, processing of the residence status, language and cultural barriers. These factors have an impact on mental health and may further worsen mental health symptoms. When compared to a previous study of shorter integration phases [19], our findings suggested a higher proportion of refugees with residence permits (97% vs. 42%) - this has been found to significantly benefit mental health in refugees, corresponding to their phase of integration [50]. Additionally, a higher proportion were employed (52% vs. 2%). As migrants spend more time in the host country and gain residence status they benefit from access to adequate healthcare, learning the local language and culture, all of which facilitate integration and finding employment. Another important factor for these refugees is that there is no violent conflict in Switzerland, and therefore this country offers a relatively good environment for mental health recovery. In our study, 51% of the population were married, compared to 31% in the study conducted by Chernet et al. [19]. The presence of a close partner can provide much needed psychological support when settling into a new environment. In addition, high resilience is associated with good mental health outcomes. The proportion of participants with high resilience scores in our study was much higher than in the Chernet et al. [19] study (70% vs. 60%). All these factors could further explain the higher frequency of symptoms of anxiety, depression, PTSD, and harmful alcohol use observed in the previous study [19].

The estimated prevalence of somatic symptom disorder in the general adult population is 5%–7% [51]. Somatic symptom disorders–i.e., when a person exerts such a strong focus on physical symptoms, that this results in major distress and/or problems functioning–are more prevalent in women in the general public, with an estimated female-to-male ratio of 10:1 [52]. This was true for our study as well with 29% of women reporting moderate to severe symptoms of somatic disorder versus 11% of men. The proportion of females in our study was much higher than that of Chernet et al. (39% vs. 11%), and the proportion of symptoms of somatic disorders was also higher (18% vs. 10%). The higher proportion of women in our study could explain the higher frequency of somatic symptoms [19].

Current evidence indicates that substance use among refugees appears to depend on the reason for migration as well as global and regional influences such as religious and cultural norms in both the country of origin and the destination country [21]. Furthermore, harmful alcohol or substance use has been documented in forced migrants and may be a coping mechanism for underlying trauma experienced during migration or related to living in a culture where alcohol is readily available, and its use is socially acceptable. The proportion of heavy episodic drinkers (harmful alcohol use) among the general population aged 15 years and above in Eritrea, Somalia and Switzerland are as follows: 5.6%, 0.3% and 36.2%, respectively [22]. A recent review indicated that hazardous/harmful alcohol use in refugees varied from 4% to 7% in community settings to 17%–36% in camp settings [21]. Our study results, which found harmful alcohol use of 12%, are slightly higher than that for community settings and the Eritrean, and Somali general population [22].

This study revealed a profile of elevated frequencies of somatic symptoms and harmful alcohol use among refugees after a relatively long stay in Switzerland. A study on the mental health of migrants, including undocumented migrants living in Geneva, Switzerland for ≥5 years revealed a profile of prevalent anxiety symptoms (36%), depression (45%), and sleep disturbance (23%) [20]. Though not directly comparable to our study due to different migrant populations and screening methods, it appears that a trend towards clinically relevant mental health symptoms may persist years after resettlement.

This study has several strengths and limitations. It is among the few studies investigating the symptoms of mental health conditions in long-term Eritrean and Somali refugee residents in the German speaking part of Switzerland. Recruiting migrants into health research is inherently challenging and low response rates are very common in such studies [53–57], leading to potential nonresponse bias- a limitation also relevant to this study. Many community members who were approached declined to participate, citing lack of time due to work commitments or childcare as well as mistrust of institutions and prior experiences of discrimination, well-documented barriers to migrant participation [53–55]. Because recruitment was conducted by community members approaching potential participants in public spaces, interactions were intentionally informal and conversational. When individuals declined participation, recruiters respected their decision without seeking further explanation or personal information. In this context, collecting data on non-responders would have been intrusive and risked eroding the trust that researchers had worked to establish within the community. Consequently, reasons for refusal were not systematically recorded and the potential influence of self-selection bias on prevalence estimates remains unknown. However, informal observations during recruitment suggest that some of those who declined to participate—particularly individuals expressing mistrust or those overwhelmed by work and childcare demands—may be underrepresented in our sample. Because such stressors are often linked to elevated psychological distress, their absence, and the resulting non-response bias, may have resulted in an underestimation of proportion of mental health symptoms in this population. Recruitment of this population required significant time, commitment, resources, and flexibility. Nevertheless, despite these challenges—and the added constraints of the COVID-19 pandemic—a considerable number of refugees agreed to participate, with a balanced distribution of males and females across age groups. The sample size was modest, particularly for Somali participants, and the number of refusals limits the generalizability of the findings. Finally, reliance on self-report questionnaires introduces the possibility of recall bias, which may also have contributed to underreporting of mental health symptoms.

In conclusion, we found that a significant proportion of Eritrean and Somali refugees experienced traumatic events during migration. Most of these refugees had been living for several years in Switzerland and had low frequencies of symptoms of PTSD, anxiety, depression and high resilience scores. However, they had elevated frequencies of symptoms of somatic disorders and harmful alcohol use. Trauma experienced during migration, post migration stressors and triggers of the traumatic experience during residence in the host country can have prolonged impact on mental health. Nevertheless, our results taken together with the existing literature, suggest that mental health conditions in refugees evolve over time, initially characterized by PTSD, anxiety and depression, and later shifting towards somatic disorders and harmful alcohol consumption. Our study highlights the need for long-term mental healthcare beyond the time of arrival in Switzerland to guarantee adequate health of the refugees. This study contributes to the understanding of refugee mental health dynamics after resettlement, providing evidence that the profile of mental health issues changes over time.
