# Supplementary material for: Mental Health and Resilience in Eritrean and Somali Refugees in Switzerland: A Cross-Sectional Study
Source: Int J Public Health. 2026 Mar 11;71:1608308. doi: 10.3389/ijph.2026.1608308 (PMC13013081; doi:10.3389/ijph.2026.1608308)
Supplement: Supplementary file 1 [file Table1.docx]

**TABLE S1**. Mental health symptoms, alcohol use and resilience in Eritrean and Somali refugees in Switzerland, stratified by sex.

|  | Sex | | |
| --- | --- | --- | --- |
|  | Male | Female |  |
| N | 62 (61%) | 40 (39%) |  |
| Experience a traumatic event n=100 |  |  |  |
| Yes | 17 (28%) | 15 (38%) |  |
| No | 44 (72%) | 24 (62%) |  |
| History of imprisonment during migration n=99 |  |  |  |
| Yes | 30 (49%) | 10 (26%) |  |
| No | 31 (51%) | 28 (74%) |  |
| Other difficulties during migration? n=99 |  |  |  |
| Yes | 7 (11%) | 3 (8%) |  |
| No | 54 (89%) | 35 (92%) |  |
| Imprisonment/difficulties during migration n=99 |  |  |  |
| Yes | 33 (54%) | 12 (32%) |  |
| No | 28 (46%) | 26 (68%) |  |
| **Somatic disorder** |  |  |  |
| PHQ-15 score [median (IQR)] | 3 (0-4) | 6 (1-12) |  |
| PHQ-15 categories [n (%)] n=97 |  |  |  |
| 0-4 none to minimal | 48 (80%) | 17 (46%) |  |
| 5-9 mild | 5 (8%) | 9 (24%) |  |
| 10-14 moderate | 5 (8%) | 9 (24%) |  |
| ≥15 severe | 2 (3%) | 2 (5%) |  |
| **Resilience** |  |  |  |
| RS-14 score [median (IQR)]: | 86 (79-90) | 88 (81-91) |  |
| RS-14 categories [n (%)] n=99 |  |  |  |
| 45-64 low resilience | 2 (3%) | 1 (3%) |  |
| 65-81 moderate resilience | 18 (30%) | 9 (24%) |  |
| ≥ 82 high resilience | 41 (67%) | 28 (73%) |  |
| **Alcohol consumption** |  |  |  |
| Have you ever consumed alcohol? n=100 |  |  |  |
| Yes | 37 (61%) | 13 (33%) |  |
| No | 24 (39%) | 26 (68%) |  |
| AUDIT score [median (IQR)]: | 2 (0-6) | 0 (0-1) |  |
| AUDIT categories [n (%)] n= 100 |  |  |  |
| 0 no risk | 24 (39%) | 26 (67%) |  |
| 1-7 low risk | 25 (41%) | 13 (33%) |  |
| 8-15 risk/hazardous | 11 (18%) | 0 (0%) |  |
| 16-19 high risk/harmful | 1 (2%) | 0 (0%) |  |
| ≥ 20 possible alcohol dependence | 0 | 0 |  |

IQR: interquartile ranges PHQ: patient health questionnaire, RS-14: resilience scale questionnaire, AUDIT: alcohol use disorder identification test. Post-traumatic stress disorder, anxiety and depression were not included as frequency of clinically relevant symptoms were too low. Mental health symptoms in Eritrean and Somali refugees, Switzerland, 2022-2023.

**TABLE S2**. Mental health symptoms, alcohol use and resilience in Eritrean and Somali refugees in Switzerland, stratified by age.

|  | Age (years) | | |
| --- | --- | --- | --- |
|  | < 35 | ≥ 35 |  |
| N | 54 (53%) | 48 (47%) |  |
| Experience a traumatic event n=100 |  |  |  |
| Yes | 15 (28%) | 17 (36%) |  |
| No | 38 (72%) | 30 (64%) |  |
| History of imprisonment during migration n=99 |  |  |  |
| Yes | 17 (33%) | 23 (49%) |  |
| No | 35 (67%) | 24 (51%) |  |
| Other difficulties during migration? n=99 |  |  |  |
| Yes | 7 (13%) | 3 (6%) |  |
| No | 45 (87%) | 44 (94%) |  |
| Imprisonment/difficulties during migration n=99 |  |  |  |
| Yes | 21 (40%) | 24 (51%) |  |
| No | 31 (60%) | 23 (49%) |  |
|  |  |  |  |
| **Somatic disorder** |  |  |  |
| PHQ-15 score [median (IQR)] | 3 (1-8) | 3 (0-9) |  |
| PHQ-15 categories [n (%)] n= 97 |  |  |  |
| 0-4 none to minimal | 34 (65%) | 31 (69%) |  |
| 5-9 mild | 11 (21%) | 3 (7%) |  |
| 10-14 moderate | 5 (10%) | 9 (20%) |  |
| ≥15 severe | 2 (4%) | 2 (4%) |  |
| **Resilience** |  |  |  |
| RS-14 score [median (IQR)]: | 88 (81-90) | 86 (78-91) |  |
| RS-14 categories [n (%)] n=99 |  |  |  |
| 45-64 low resilience | 1 (2%) | 2 (4%) |  |
| 65-81 moderate resilience | 13 (25%) | 14 (30%) |  |
| ≥ 82 high resilience | 38 (73%) | 31 (66%) |  |
| **Alcohol consumption** |  |  |  |
| Have you ever consumed alcohol? n=100 |  |  |  |
| Yes | 27 (51%) | 23 (49%) |  |
| No | 26 (49%) | 24 (51%) |  |
| AUDIT score [median (IQR)]: | 1 (0-3) | 0 (0-3) |  |
| AUDIT categories [n (%)] n= 100 |  |  |  |
| 0 no risk | 26 (49%) | 24 (51%) |  |
| 1-7 low risk | 21 (40%) | 17 (36%) |  |
| 8-15 risk/hazardous | 5 (9%) | 6 (13%) |  |
| 16-19 high risk/harmful | 1 (2%) | 0 (0%) |  |
| ≥ 20 possible alcohol dependence | 0 | 0 |  |

IQR: interquartile ranges PHQ: patient health questionnaire, RS-14: resilience scale questionnaire, AUDIT: alcohol use disorder identification test. Post-traumatic stress disorder, anxiety and depression were not included as frequency of clinically relevant symptoms were too low. Mental health symptoms in Eritrean and Somali refugees, Switzerland, 2022-2023.

**TABLE S3**. Mental health symptoms, alcohol use and resilience in Eritrean and Somali refugees in Switzerland, stratified by imprisonment or other difficulties during migration.

|  | Imprisonment or other difficulties during migration | |
| --- | --- | --- |
|  | Yes | No |
| N | 45 (46%) | 54 (54%) |
| **Somatic disorder** |  |  |
| PHQ-15 score [median (IQR)] | 3 (1-8) | 3 (0-7) |
| PHQ-15 categories [n (%)] n=97 |  |  |
| 0-4 none to minimal | 27 (61%) | 38 (72%) |
| 5-9 mild | 9 (20%) | 5 (9%) |
| 10-14 moderate | 6 (14%) | 8 (15%) |
| ≥15 severe | 2 (5%) | 2 (4%) |
| **Resilience** |  |  |
| RS-14 score [median (IQR)]: | 89 (82-91) | 85 (77-89) |
| RS-14 categories [n (%)] n=99 |  |  |
| <64 low resilience | 0 (0.0%) | 3 (6%) |
| 65-81 moderate resilience | 9 (20%) | 18 (33%) |
| ≥ 82 high resilience | 36 (80%) | 33 (61%) |
| **Alcohol consumption** |  |  |
| Have you ever consumed alcohol? n=99 |  |  |
| Yes | 24 (53%) | 25 (46%) |
| No | 21 (47%) | 29 (54%) |
| AUDIT score [median (IQR)]: | 1 (0-3) | 0 (0-2) |
| AUDIT categories [n (%)] n=99 |  |  |
| 0 no risk | 21 (47%) | 29 (54%) |
| 1-7 low risk | 18 (40%) | 19 (35%) |
| 8-15 risk/hazardous | 6 (13%) | 5 (9%) |
| 16-19 high risk/harmful | 0 (0%) | 1 (2%) |
| ≥ 20 possible alcohol dependence | 0 | 0 |

IQR: interquartile ranges PHQ: patient health questionnaire, RS-14: resilience scale questionnaire, AUDIT: alcohol use disorder identification test. Post-traumatic stress disorder, anxiety and depression were not included as frequency of clinically relevant symptoms were too low. Mental health symptoms in Eritrean and Somali refugees, Switzerland, 2022-2023.
